# Supplementary material for: Reconciling Mining with the Conservation of Cave Biodiversity: A Quantitative Baseline to Help Establish Conservation Priorities
Source: PLoS One. 2016 Dec 20;11(12):e0168348. doi: 10.1371/journal.pone.0168348 (PMC5173368; doi:10.1371/journal.pone.0168348)
Supplement: S1 Dataset — (ZIP) [file pone.0168348.s002.zip › Taxa/Serra Norte/SN_2013/N4WS/Lista N4WS-34.pdf]

CAVIDADE N4WS-0034

Classe	Ordem	Fam/Outros	Gên/Outros	Espécie	S-U	U-S	Zon.
Arachnida	Acari	(Parasitengonina)		sp.2	X		E
Arachnida	Acari	Neothyridae	*Diplothyrus*	*schubarti*		X	E
Arachnida	Amblypygi	Charinidae	*Charinus*	sp.1		1	P
Arachnida	Amblypygi	Phrynidae	*Heterophrynus*	*longicornis*	3	1	E/P
Arachnida	Araneae	Ochyroceratidae	*Ochyrocera*	sp.1		X	E
Arachnida	Araneae	Pholcidae	*Leptopholcus*	sp.1	X	X	E/P
Arachnida	Araneae	Pholcidae	*Mesabolivar*	*eberhard*	X		E
Arachnida	Araneae	Pholcidae	Ninetinae	sp.1		X	E
Arachnida	Araneae	Theraphosidae		jovem		1	E
Arachnida	Araneae	Theridiosomatidae		jovem	X		E
Arachnida	Ricinulei	Ricinoididae	*Cryptocellus*	*tarsilae*		X	P
Entognatha	Collembola	Isotomidae		sp.1	X		E
Entognatha	Collembola	Paronellidae		sp.1	X		E
Entognatha	Collembola	Paronellidae		sp.4	X	X	P
Entognatha	Collembola	Symphypleona		sp.1	X		E
Entognatha	Diplura	Campodeidae		sp.1	X		P
Insecta	Blattodea			jovem	1	1	E/P
Insecta	Coleoptera	Carabidae		sp.3	X		E
Insecta	Coleoptera	Dytiscidae		sp.12A		X	E
Insecta	Coleoptera	Dytiscidae		sp.B		X	P
Insecta	Diptera	Cecidomyiidae	Cecidomyiinae	sp.	X	X	E/P
Insecta	Diptera	Ceratopogonidae		jovem	X		E/P
Insecta	Diptera	Culicidae	*Culex*	sp.	X	X	P
Insecta	Diptera	Tipulidae	Tipulinae	sp.	X		E
Insecta	Heteroptera	Veliidae	*Paravelia*	sp.1	X	X	E
Insecta	Homoptera	Cixiidae		jovem		X	P
Insecta	Hymenoptera	Formicidae	*Camponotus*	*atriceps*		1	E
Insecta	Hymenoptera	Formicidae	*Nylanderia*	sp.3		X	P
Insecta	Hymenoptera	Formicidae	*Odontomachus*	sp.1		1	E
Insecta	Hymenoptera	Formicidae	*Pachycondyla*	*striata*	X		E/P
Insecta	Hymenoptera	Formicidae	*Pheidole*	sp.2	X		E
Insecta	Hymenoptera	Formicidae	*Wasmannia*	sp.1		X	E/P
Insecta	Lepidoptera	Gelechioidea		sp.4		X	E
Insecta	Lepidoptera	Noctuoidea		sp.1		1	E
Insecta	Lepidoptera			jovem	X		E
Insecta	Neuroptera	Myrmeleontidae		jovem		1	E
Insecta	Orthoptera	Phalangopsidae	*Paraclodes*	sp.	4		E
Insecta	Orthoptera	Phalangopsidae	*Phalangopsis*	sp.	14		E/P
Insecta	Psocoptera	Psocomorpha		jovem	X		E
Malacostraca	Isopoda	Philosciidae		sp.2	X	X	E/P
Turbellaria	Tricladida			sp.1		X	E
Amphibia	Anura	Strabomantidae	*Pristimantis*	cf. *fenestratus*	3		E/P
Mammalia	Chiroptera	Emballonuridae	*Peropteryx*	sp.	5	5	E/P
